# Ageing has no effect on the regulation of the ubiquitin proteasome-related genes and proteins following resistance exercise

**DOI:** 10.3389/fphys.2014.00030

**Published:** 2014-01-31

**Authors:** Renae J. Stefanetti, Evelyn Zacharewicz, Paul Della Gatta, Andrew Garnham, Aaron P. Russell, Séverine Lamon

**Affiliations:** Centre for Physical Activity and Nutrition, School of Exercise and Nutrition Sciences, Deakin UniversityBurwood, VIC, Australia

**Keywords:** skeletal muscle, resistance exercise, muscle protein breakdown, ubiquitin-proteasome, atrogene signaling

## Abstract

Skeletal muscle atrophy is a critical component of the ageing process. Age-related muscle wasting is due to disrupted muscle protein turnover, a process mediated in part by the ubiquitin proteasome pathway (UPP). Additionally, older subjects have been observed to have an attenuated anabolic response, at both the molecular and physiological levels, following a single-bout of resistance exercise (RE). We investigated the expression levels of the UPP-related genes and proteins involved in muscle protein degradation in 10 older (60–75 years) vs. 10 younger (18–30 years) healthy male subjects at basal as well as 2 h after a single-bout of RE. MURF1, atrogin-1 and FBXO40, their substrate targets PKM2, myogenin, MYOD, MHC and EIF3F as well as MURF1 and atrogin-1 transcriptional regulators FOXO1 and FOXO3 gene and/or protein expression levels were measured via real time PCR and western blotting, respectively. At basal, no age-related difference was observed in the gene/protein levels of atrogin-1, MURF1, myogenin, MYOD and FOXO1/3. However, a decrease in FBXO40 mRNA and protein levels was observed in older subjects, while PKM2 protein was increased. In response to RE, *MURF1*, atrogin-1 and *FBXO40* mRNA were upregulated in both the younger and older subjects, with changes observed in protein levels. In conclusion, UPP-related gene/protein expression is comparably regulated in healthy young and old male subjects at basal and following RE. These findings suggest that UPP signaling plays a limited role in the process of age-related muscle wasting. Future studies are required to investigate additional proteolytic mechanisms in conjunction with skeletal muscle protein breakdown (MPB) measurements following RE in older vs. younger subjects.

## Introduction

Maintaining skeletal muscle mass is a critical component of health and sustaining life. Age-related skeletal muscle wasting is a preferential loss of type II muscle fibers (Lexell and Taylor, 1991; Lexell, 1995; Kadi et al., 2004; Verdijk et al., 2007), resulting in a progressive decline in muscle mass, strength and function (Evans, 1995b; Deschenes, 2004). On average, 0.5–1.0% of muscle mass is lost per year from 40 years of age, with the loss of muscle mass rapidly increasing after 65 years of age (Greenlund and Nair, 2003). Age-related muscle wasting reduces functional independence (Cooper, 1997) and increases the risk of falls, chronic metabolic disease (obesity, type 2 diabetes) (Park et al., 2006) and ultimately, mortality (Mahoney et al., 1994; Newman et al., 2006). A better understanding of the molecular mechanisms involved in the decline of muscle mass as we age is therefore required to aid the development of effective countermeasures to prevent, attenuate and/or reverse this process.

In healthy individuals, muscle mass is maintained via the finely regulated balance existing between muscle protein synthesis (MPS) and muscle protein breakdown (MPB). An increase in muscle mass is largely due to an increase in the size of pre-existing muscle fibers via net accretion of muscle sarcoplasmic and myofibrillar proteins (Goldberg, 1969; Roman et al., 1993; Staron et al., 1994; Mccall et al., 1996). In contrast, the ubiquitin proteasome pathway (UPP) is one of the major mechanisms responsible for human MPB (Passmore and Barford, 2004; Sandri, 2013). The muscle-specific E3-ubiquitin ligases, muscle ring finger-1 (MURF1) and atrogin-1 [muscle atrophy F-box (MAFbx), FBXO32] (Bodine et al., 2001; Gomes et al., 2001) are transcriptionally regulated by Forkhead box O (FOXO) family members, FOXO1 and FOXO3 (Brunet et al., 1999; Sandri et al., 2004; Stitt et al., 2004). Known substrate targets of MURF1 include pyruvate kinase M2 (PKM2) (Hirner et al., 2008) and myosin heavy chain (MHC) (Clarke et al., 2007; Fielitz et al., 2007; Cohen et al., 2009), whereas known substrate targets of atrogin-1 include elongation initiation factor 3 subunit 5 (EIF3F) (Lagirand-Cantaloube et al., 2008), MYOD (Tintignac et al., 2005) and myogenin (MYOG) (Jogo et al., 2009). These substrate targets are proteins involved in biological processes essential to muscle health and function, such as MPS (EIF3F), (Lagirand-Cantaloube et al., 2008), development and regeneration (MYOD and MYOG) (Tintignac et al., 2005; Jogo et al., 2009), metabolism (PKM2) (Hirner et al., 2008) as well as structure and contraction (MHC) (Clarke et al., 2007; Fielitz et al., 2007; Cohen et al., 2009).

Resistance exercise (RE) training increases muscle mass, strength and function (Roman et al., 1993; Staron et al., 1994; Hortobagyi et al., 1996; Mccall et al., 1996; Wernbom et al., 2007) and is often used as an intervention to improve muscle functional capacity (Evans, 1995a). The principal response after a single-bout of RE is a rapid (within 2–4 h) transient increase in MPS, particularly in myofibrillar MPS (Phillips et al., 1997). However, MPB also increases following RE, albeit to a lesser extent and of a shorter-lived duration than MPS (Biolo et al., 1995; Phillips et al., 1997). While the aetiology of aged-related muscle atrophy is multifactorial, a disruption in the regulation of skeletal muscle protein turnover seems to play a major role (Boirie, 2009). Despite early reports of a large decrease in the basal rate of MPS (Welle et al., 1993, 1995; Balagopal et al., 1997; Hasten et al., 2000) and a large increase in the basal rate of MPB (Trappe et al., 2004) in older compared to younger individuals, recent studies demonstrate little or no significant difference in basal MPS and MPB between older vs. younger individuals (Volpi et al., 1999, 2000, 2001; Hasten et al., 2000; Paddon-Jones et al., 2004; Cuthbertson et al., 2005; Katsanos et al., 2005, 2006; Rasmussen et al., 2006; Kumar et al., 2009). RE-induced stimulation of MPS occurs in both younger and older individuals (Yarasheski et al., 1993; Welle et al., 1994; Volpi et al., 1999; Hasten et al., 2000; Sheffield-Moore et al., 2004), but with an attenuated magnitude in older individuals (Kumar et al., 2009); a phenomenon referred to as anabolic resistance. However, the regulation of MPB in older vs. younger individuals in response to RE and how this may contribute to anabolic resistance is not well defined. In particular, the gene and protein expression pattern of the members of the UPP pathway at rest and following RE in older and younger individuals has received little attention to date. Such investigations will provide a better understanding of the activation and regulation of the UPP pathway in older vs. younger individuals. A greater activation of the proteolytic markers in older subjects may indicate an increase in MPB; a phenomenon that might therefore contribute to the attenuated MPS response characteristic of anabolic resistance.

Conflicting results have been observed in the age-related mRNA regulation of *MURF1* and atrogin-1 (*FBXO32*), suggestive of gender, muscle type and species influences (rodent vs. human muscle). Compared to younger rodent muscle, studies have shown that baseline *MURF1* and atrogin-1 mRNA levels in aged rodents increase in the tibialis anterior (Clavel et al., 2006), decrease in the gastrocnemius muscle (Edstrom et al., 2006) or do not differ in the extensor digitorum longus and soleus muscle (Gaugler et al., 2011). In humans, some studies find an increase in *MURF1* baseline mRNA expression in older muscle compared to younger muscle (Raue et al., 2007; Dalbo et al., 2011; Merritt et al., 2013), while other groups report no differences (Welle et al., 2003; Whitman et al., 2005; Léger et al., 2008; Greig et al., 2011; Fry et al., 2013) Albeit one study showing a subtle elevation in baseline atrogin-1 mRNA expression with ageing (Merritt et al., 2013), age-related differences in basal atrogin-1 mRNA expression do not occur (Welle et al., 2003; Whitman et al., 2005; Raue et al., 2007; Léger et al., 2008; Dalbo et al., 2011; Greig et al., 2011; Fry et al., 2013; Sandri et al., 2013). In response to single-bout RE in human muscle, *MURF1* mRNA increases in both younger and older subjects 3–6 h post-RE (Raue et al., 2007; Fry et al., 2013). Atrogin-1 mRNA is either unchanged between older vs. younger individuals (Fry et al., 2013), or increases 4 h post-single-bout RE in older individuals only (Raue et al., 2007). Although the effect of RE on MURF1 and atrogin-1 expression has been well described (for review see Russell, 2010), whether the protein levels of muscle-specific E3-ubiquitin ligases as well as their substrate targets are differentially altered in younger vs. older individuals in human skeletal muscle in response to RE is yet to be investigated.

Therefore, the aim of the current study was to report the gene and protein expression patterns of MURF1, atrogin-1 and FBXO40, a gene encoding another muscle specific F-box protein (Ye et al., 2007), the substrate targets PKM2, myogenin, MYOD, MHC and EIF3F as well as MURF1 and atrogin-1 transcriptional regulators FOXO1 and FOXO3 in older vs. younger individuals at basal and in response to a single-bout of RE following overnight fasting.

## Materials and methods

### Subjects

Ten younger (18–30) and 10 older (60–75) healthy males participated in the study. The study was approved by the Deakin University Human Research Committee (#2011-043) in accordance to the *Declaration of Helsinki* (2013)^1^. All participants gave their informed consent and agreed to engage in muscle biopsies and physiological testing. The subjects were physically active but had not participated in a RE training programme within 6 months prior to the study. Exclusion criteria included any type of protein supplementation and anabolic steroids. Physiological characteristics of the subjects are summarized in Table 2.

### Dual-energy X-ray absorptiometry scan (DXA)

Total body and regional (arms and legs) body composition [lean mass (LM), fat mass (FM) and % body fat] and lumbar spine (L1-L4) and proximal femur (femoral neck and total hip) areal bone density (aBMD) were assessed via DXA (Lunar Prodigy, GE Lunar Corp., Madison WI), using software version 12.30.008.

### Preliminary testing

At least 2 weeks prior to the single-bout exercise session, the subjects were familiarized with the equipment (Nautilus Leg Extension, Fitness Generation, Rowville, Australia) and correct lifting technique. Their one-repetition-maximum (1 RM) was determined using a 5 RM test for leg extension exercise. Estimated 1 RM was then calculated using the Brzycki equation:

1RM =weight lifted (kg) /1.0278−[reps to fatigue × 0.0278]

(Nascimento et al., 2007).

### Muscle biopsies

Skeletal muscle samples were obtained under local anesthesia (1% Xylocaine) from the belly of the vastus lateralis muscle using a percutaneous needle biopsy technique (Bergstrom, 1962) modified to include suction (Evans et al., 1982). Following an incision through the skin, muscle biopsies were taken using a Bergstrom needle. The muscle samples were immediately frozen in liquid nitrogen and used for RNA and protein extraction.

### Single-bout exercise protocol

Subjects had been instructed to abstain from strenuous exercise, caffeine and alcohol consumption for 24 h prior to the trial. One night before the trial, the subjects were instructed to consume a provided standardized meal containing 20% fat, 14% protein and 66% carbohydrate. The subjects arrived after an overnight fast and rested in the supine position for 2 h prior to the sampling of the 1st muscle biopsy. Immediately following the muscle biopsy, subjects completed a 3-min light cycling warm-up followed by a leg extension exercise session. The single-bout exercise protocol consisted of three sets of 14 repetitions at 60% of maximal voluntary contraction (60% 1 RM) with 2-min recovery between sets. Immediately following this, subjects completed another 3-min light cycling exercise, and were thereafter instructed to rest in the supine position again. Two hours post-exercise, another muscle biopsy was taken from the opposite leg to avoid any local effect of the pre-exercise biopsy.

### Protein extraction and western blotting

**Figure A1:**
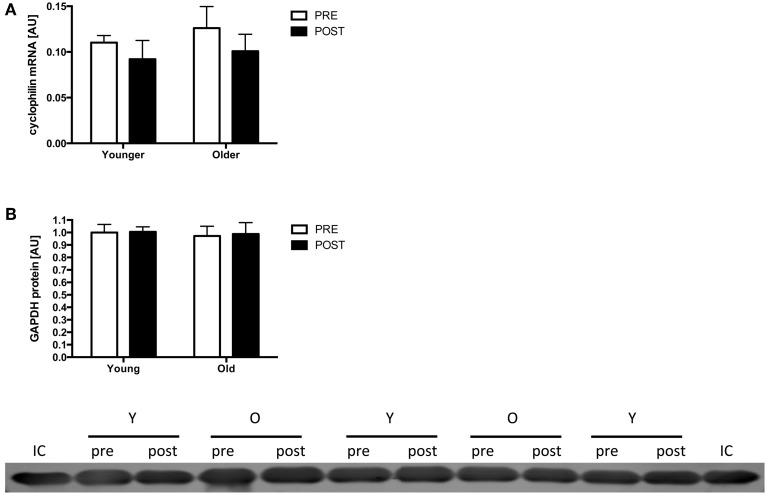
**(A)** Cyclophilin mRNA expression (AU), **(B)** GAPDH protein expression (AU) and representative blot for GAPDH protein analysis. IC, internal control; Y, young; O, old.

**Figure A2:**
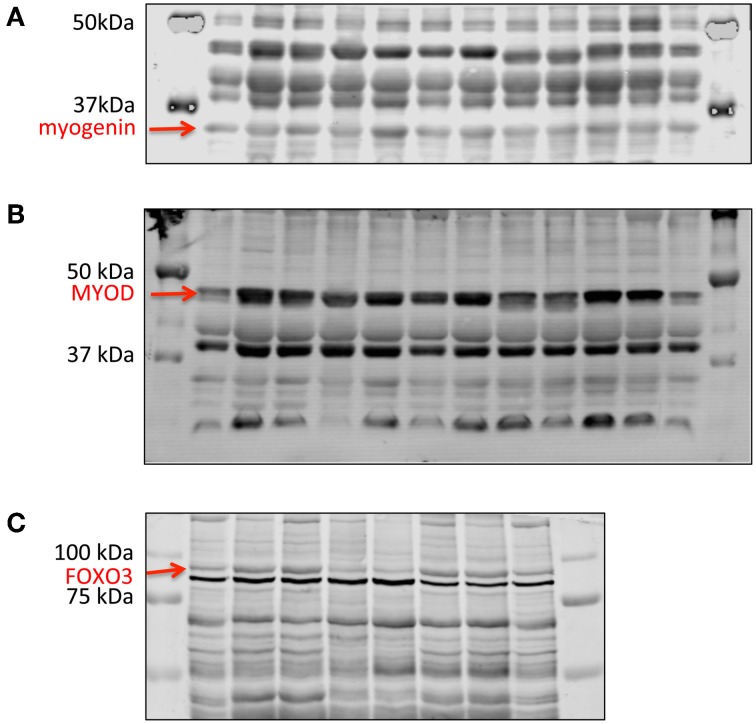
**Representative western blots for the proteins myogenin (A), MYOD (B), and FOXO3 (C)**.

Total protein from whole tissue lysates was extracted using RIPA buffer (Millipore, North Ryde, Australia) with 1 μ L/mL protease inhibitor cocktail (Sigma, Castle Hill, Australia) and 10 μ L/mL Halt Phosphatase Inhibitor Single-Use Cocktail (Thermo Scientific, Rockford, USA). Total protein content was determined using the BCA Protein Assay Kit (Pierce Biotechnology, Rockford, USA) according to the manufacturer's instructions. Proteins were separated by SDS-polyacrylamide gel (PAGE) in a buffer containing 12 mM Tris-HCl (pH 8.8), 200 mM glycine and 0.1% SDS. Proteins were transferred onto an Immobilon-FL PDVF membrane (Millipore, Billerica, MA) in a Bjerrum buffer containing 50 mM Tris, 17 mM glycine and 10% methanol. Membranes were blocked with 5% BSA in PBS for 1 h at room temperature and were thereafter incubated at 4°C overnight with the following primary antibodies diluted in 5% BSA in PBS: MURF1 (MP3401, ECM Biosciences, Versailles, KY) at 1:1000; FBXO40 (H00051725-B01P, Abnova, Taipei City, Taiwan) at 1:200; FOXO1 (C29H4, Cell Signaling Technology, Danvers, MA) at 1:500; and FOXO3 (ab17026, Abcam, Cambridge, MA, USA) at 1:500; EIF3F (Jomar Bioscience, Adelaide, Australia) at 1:500; MYOD (M-318: sc-760, Santa Cruz Biotechnology) at 1:200; myogenin (MAB3876, Merck Millipore, Billerica, MA) at 1:300; PKM2 (3198, Cell Signaling Technology, Danvers, MA) at 1:1000; and MHC/ sarcomeric myosin (MF 20, Developmental Studies Hybridoma Bank, Iowa City, IA) at 1:1000. Alternatively, membranes were blocked with 5% BSA in TBST for 1 h at room temperature and were thereafter incubated at 4°C overnight with the following primary antibodies: phospho-FOXO1 (Ser256, 9461, Cell Signaling Technology, Danvers, MA) at 1:500; and phospho-FOXO3 (Ser253, 9466, Cell Signaling Technology, Danvers, MA) at 1:400. FOXO1 and FOXO3 can be activated through phosphorylation by Akt at Ser256 and Ser253, respectively, resulting in their nuclear export and inhibition of transcription factor activity. Following overnight primary antibody incubation, membranes were washed with either PBS or TBST (4 × 5 min) and were subsequently incubated for 1 h with the following infrared-fluorescent conjugated secondary antibodies, diluted at 1:5000 in PBS or TBST containing 50% Odyssey® Blocking Buffer (LI-COR Biosciences, Lincoln, USA) and 0.01% SDS: IRDye 800CW goat anti-rabbit IgG (LI-COR Biosciences, Lincoln, USA) for MURF1, FOXO1, phospho-FOXO1, phospho-FOXO3, EIF3F, MYOD and PKM2; IRDye 800CW donkey anti-goat IgG (LI-COR Biosciences, Lincoln, USA) for FOXO3; and Alexa Fluor® 680 rabbit anti-mouse IgG (Invitrogen, Carlsbad, CA) for FBXO40, myogenin, MHC and GAPDH. After washing, the proteins were exposed on an Odyssey® Infrared Imaging System (LI-COR Biosciences, Lincoln, USA) and individual protein band optical densities were determined using the Odyssey® Image Studio Lite Version 3.3.4. All blots were normalized against the GAPDH protein (G8795; Sigma-Aldrich, Sydney, Australia) (see Figure A1B). Extended western blot pictures for the proteins myogenin, MYOD and FOXO3 are represented in Figure A2.

### RNA extraction and reverse transcription

RNA was extracted from ~15 mg of skeletal muscle samples using Tri-Reagent® Solution (Ambion Inc., Austin, TX, USA) according to the manufacturer's protocol. The RNA concentration was assessed using the Nanodrop 1000 Spectrophotometer (Thermo Fisher Scientific, MA, USA). The ratio between A260/A280 was 1.75–1.95 for all samples. First-strand cDNA was generated from 1 μ g RNA in 20 μ l reaction buffer using the High Capacity RT-kit (Applied Biosystems, Carlsbad, CA, USA), 1 × RT buffer and random primers, 8 mM dNTP and 2.5 U μ l^−1^ MultiScribeTM RT enzyme. The RT protocol consisted of 10 min at 25°C, 120 min at 37°C, 5min at 85°C then cooled to 4°C. The cDNA was stored at −20°C until further analysis.

### Real-time PCR

Real-time PCR (RT-PCR) was carried out using a Stratagene MX3000 thermal cycler to measure mRNA levels. mRNA levels for atrogin-1, MURF1, FX040, FOXO1 and FOXO3 were measured using 1 × SYBR® Green PCR MasterMix (Applied Biosystems, Carlsbad, CA, USA) and 5 ng of cDNA. To compensate for variations in input RNA amounts and efficiency of the reverse transcription, data were normalized to cyclophilin (see Figure A1A). All primers were used at a final concentration of 300 nM and probes at 100 nM. Primer details are provided in Table 1. The PCR conditions were 1 cycle of 10 min at 95°C; 40 cycles of 30 s at 95°C; 60 s at 60°C. For cyclophilin, a melting curve was included at the end of the PCR cycles. RT-PCR analyses were conducted in triplicate. Ct values were obtained from the MxPro QPCR software (Agilent Technologies, Santa Clara, CA, USA). Mean Ct values and their standard error of the mean (s.e.m.) were calculated for each of the samples. Ct values were logarithmically transformed and mean log transformed Ct values (referred to as arbitrary unit values) were then considered for further analysis.

**Table 1:** **PCR human primer sequences**.

**Gene**	**GenBank accession number**	**Sequences**
*MURF1*	NM_032588.3	Forward CCTGAGAGCCATTGACTTTGG
		Reverse CTTCCCTTCTGTGGACTCTTCCT
		Probe (Texas Red)-AGGAAGAATTCATTGAAGAAGAAGATCAGG(BHQ-2)
*FBXO32* (Atrogin-1)	NM_058229.3	Forward GCAGCTGAACAACATTCAGATCAC
		Reverse CAGCCTCTGCATGATGTTCAGT
		Probe (FAM)-CTTCAAAGGCACCTTCACTGACCT G(BHQ-1)
*FBXO40*	NM_016298.3	Forward AGTCCACAGAGAGATCTG
		Reverse TGTGCTCTACAATGTTGAA
		Probe (HEX)-AGTTCAGCAGCCTCTTCTCCA(BHQ)
*FOXO1*	NM_002015	Forward AAGAGCGTGCCCTACTTCAA
		Reverse CTGTTGTTGTCCATGGATGC
*FOXO3*	NM_001455	Forward CTTCAAGGATAAGGGCGACA
		Reverse TCTTGCCAGTTCCCTCATT
*PPIA* (cyclophilin A)	NM_021130	Forward CATCTGCACTGCCAAGACTGA
		Reverse TTCATGCCTTCTTTCACTTTGC

*MuRF1, muscle ring finger-1; FBXO32, F-box only protein 32/Atrogin-1, muscle atrophy F-box; FBXO40, F-box protein 40; FOXO1, forkhead transcription factor-1; FOXO3, forkhead transcription factor-3; PPIA, peptidylprolyl isomerase A, cyclophilin A*.

### Statistical methods

All data are reported as mean ± s.e.m. Unless specified differently, a Two-Way analysis of variance (ANOVA) for age and exercise was used to compare group means. Diagnostic plots of residuals and fitted values were checked to ensure homogeneity of variance (a key assumption for ANOVA). Consequently, all data were log10-transformed and analyses were conducted on these transformed scales. The least significant difference (LSD) test was used to compare pairs of means. The significance levels for both the *F*-tests in the ANOVA and the LSD tests were set at *p* < 0.05.

## Results

### Subjects' demographics

Table 2 summarizes the subjects' physiological characteristics. No significant difference in body mass, tissue composition and maximal voluntary contraction (1 RM) could be observed between the two subjects groups.

**Table 2:** **Subjects' demographics**.

	**Younger**	**Older**	***P*-value**
Age [years]	24.2 ± 0.9	66.6 ± 1.1	<0.05
Height [cm]	180.0 ± 2.0	174.60 ± 1.8	0.06
Body mass [kg]	73.8 ± 3.6	83.4 ± 7.1	0.25
Fat [kg]	13.6 ± 2.7	19.5 ± 3.9	0.23
Lean [kg]	57.9 ± 1.9	60.2 ± 3.3	0.55
BMC [kg]	3.3 ± 0.2	3.2 ± 0.2	0.67
BMI	23.3 ± 0.9	27.7 ± 1.9	0.10
Lean/total body mass	0.8 ± 0.02	0.7 ± 0.02	0.09
1 RM [kg]	98.2 ± 7.0	80.9 ± 7.2	0.1

*Values are mean ± s.e.m. BMC, bone mass content; BMI, body mass index; 1 RM, 1 repetition maximum. Statistical significance was determined by unpaired Student's t-tests*.

### Gene expression with exercise and ageing

**Figure 1:**
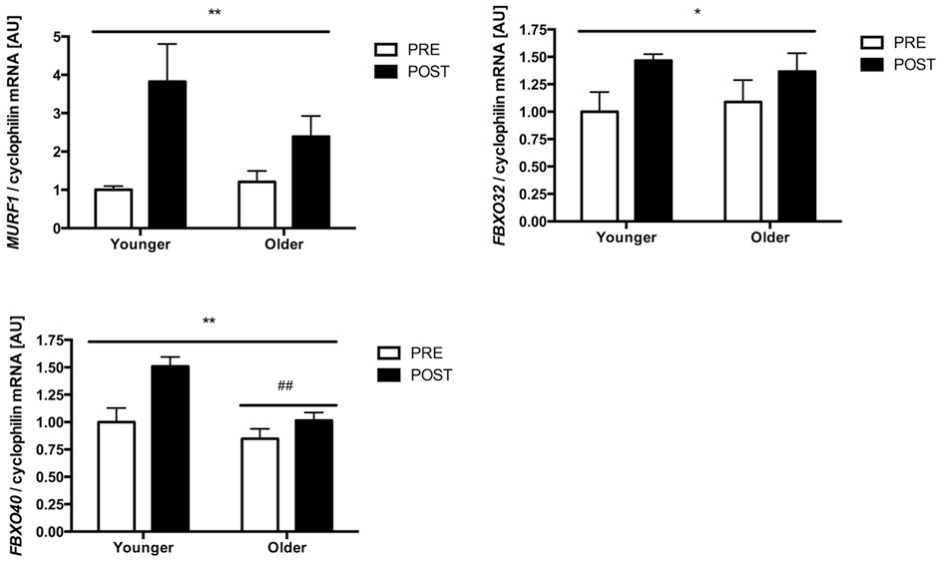
***MURF1*, atrogin-1 (*FBXO32*) and *FBXO40* mRNA expression following a single-bout of resistance exercise in younger and older people**. Note that no changes were observed for the other genes measured. ^*^significant exercise effect, *p* < 0.05, ^**^significant exercise effect, *p* < 0.01, ##significantly different from Young, *p* < 0.01. The reported statistical significance is based on analysis of the transformed data but the reported means ± s.e.m. are on the original (untransformed) scale.

In both younger and older subjects *MURF1*, atrogin-1 (*FBXO32*) and *FBXO40* mRNA levels were significantly increased 1.5-fold and 1.3-fold (*p* < 0.05), 3.8-fold and 2-fold (*p* < 0.01) and 1.5-fold and 1.2-fold (*p* < 0.01), respectively, 2 h following RE (Figure 1). In addition, *FBXO40* mRNA levels were decreased by 25% in older subjects when compared to younger subjects (*p* < 0.01). Exercise and ageing had no effect on *FOXO1* and *FOXO3* mRNA levels (data not shown). No age × exercise interaction was observed for any of the genes measured.

### Protein expression with exercise and ageing

**Figure 2:**
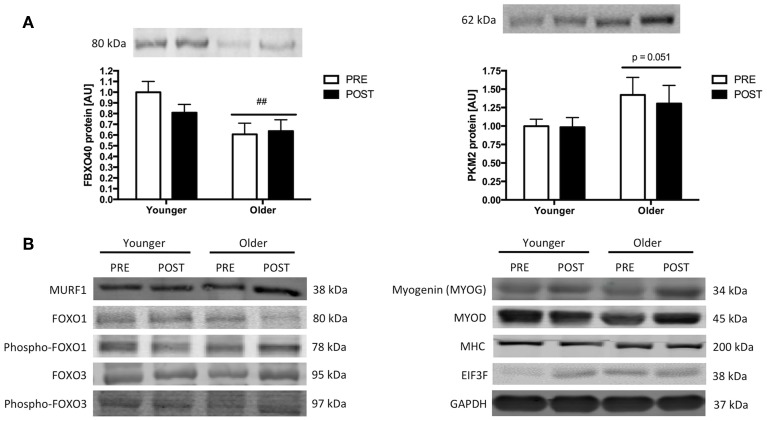
**(A)** FBXO40 and PKM2 protein expression following a single-bout of resistance exercise in younger and older people. Note that no changes were observed for the other proteins measured. ##significantly different from Young, *p* < 0.01. The reported statistical significance is based on analysis of the transformed data but the reported means ± s.e.m. are on the original (untransformed) scale. **(B)** Representative western blot images of the MURF1, total and phospho-FOXO1, total and phospho-FOXO3, myogenin (MYOG), MYOD, MHC, and EIF3F proteins measured before and after exercise. GAPDH was measured as a control for protein loading.

A single-bout of RE did not influence the expression of any of the proteins measured. In relation to the effect of age on protein expression, FBXO40 protein levels were 1.5-fold higher in younger subjects than in older subjects (*p* < 0.01). We also observed a trend (*p* = 0.051) for the effect of age on PKM2 protein levels, which were 1.6-fold higher in older subjects when compared to younger subjects. Figure 2 depicts changes in protein expression as well as representative western blot pictures for each protein measured.

## Discussion

Age-related muscle atrophy is linked to disrupted protein turnover in MPS and MPB as well as reduced regenerative capacity (Brack and Rando, 2007). When compared to younger subjects, older subjects display an impaired phosphorylation of the members of the MPS pathways at rest (Léger et al., 2008) and impaired MPS following RE (Kumar et al., 2009), a phenomenon referred to as anabolic resistance. However, how the UPP-related genes and proteins involved in MPB are regulated in older subjects in response to RE and their potential contribution to anabolic resistance is currently unknown.

Differences in the baseline expression levels of the skeletal muscle MURF1 and atrogin-1 in younger vs. older individuals is a contentious topic. This is due, at least partially, to the discrepancies existing between the studied population cohorts in terms of sex, age, physiological characteristics and level of fitness. For example, it is commonly acknowledged that the older subjects recruited on a voluntary basis for an exercise trial protocol are not representative of the average elderly population and that their average level of fitness is expected to be higher; a parameter that needs to be considered when comparing our results to others. In the present study, we failed to observe an upregulation of MURF1 mRNA or protein in older subjects when compared to younger subjects; a result in line with previous results from our group (Léger et al., 2008) and others (Welle et al., 2003; Whitman et al., 2005; Greig et al., 2011). However, it has been reported that at baseline, *MURF1* mRNA levels were increased in older men displaying a similar average age and level of fitness as our subjects (Dalbo et al., 2011), as well as in much older women (>80 years) (Raue et al., 2007) when compared to younger individuals. Consistent with previous findings in humans, no age-related difference was found in the basal level of atrogin-1 (*FBXO32*) mRNA (Welle et al., 2003; Whitman et al., 2005; Raue et al., 2007; Léger et al., 2008; Dalbo et al., 2011; Greig et al., 2011; Fry et al., 2013; Sandri et al., 2013). Similarly, no age-dependent differences were observed at baseline for FOXO1 and FOXO3 mRNA and protein or in the phosphorylation levels of FOXO1 and FOXO3 protein, the transcriptional regulators of MURF1 and atrogin-1. Baseline *FOXO3* mRNA levels were increased in much older women (>80 years) compared to younger women (Raue et al., 2007). At baseline, older subjects had a lower cytosolic FOXO3 phosphorylation and a higher total nuclear FOXO3 level compared to younger subjects (Williamson et al., 2010); potentially resulting in more transcriptional activity. However, we previously observed a decrease in nuclear FOXO1 and FOXO3 protein in sarcopenic human skeletal muscle (Léger et al., 2008). Although we did not measure nuclear proteins in this study, no differences in FOXO1 and FOXO3 phosphorylation levels were observed between younger and older subjects. This suggests that the sub-cellular localization of the FOXO proteins might not be a direct indication of their activity.

Although other protein degradation mechanisms such as the autophagy-lysosome system are unbalanced during age-related muscle atrophy, at least in rodent muscle (for review see Bonaldo and Sandri, 2013), our observations are consistent with recent work demonstrating that the basal MPB rate does not significantly differ with ageing (Volpi et al., 1999, 2000, 2001; Hasten et al., 2000; Paddon-Jones et al., 2004; Cuthbertson et al., 2005; Katsanos et al., 2005, 2006; Rasmussen et al., 2006; Kumar et al., 2009). However, gene and protein expression analysis 2 h post-exercise can not be reflective of the whole MPB process occurring following RE. Further investigations that include a post-exercise time course and direct measure of MPB obtained at similar time points are therefore required. Basic limitations of most MBP measures, including the AV balance approach, such as invasiveness, possible inclusion of non-muscle tissue or potential variations due to non-steady state conditions such as an exercise intervention should yet be taken into account. Interestingly, genetic deletion of atrogin-1 in rodents during ageing results in significant muscle atrophy and a shortened lifespan, while a reduced muscle force occurs in both MURF1 and atrogin-1 aged knockout mice (Sandri et al., 2013); suggesting that these UPP genes are essential for normal protein turnover during ageing (Sandri et al., 2013).

*MURF1*, atrogin-1 and *FBXO40* mRNA levels were all increased by exercise, with no differential effect of exercise between the age groups. MURF1 and atrogin-1 are typically regulated in response to a single-bout of RE. *MURF1* mRNA is commonly upregulated 1-4 h post-RE (Louis et al., 2007; Mascher et al., 2008; Glynn et al., 2010). In contrast, some studies observed no changes in atrogin-1 mRNA at 1–2 h post-exercise (Hulmi et al., 2009; Glynn et al., 2010) or a decreased atrogin-1 mRNA 2 h post-exercise (Reitelseder et al., 2013). Raue et al. reported an age effect in the increase of atrogin-1 mRNA, but not *MURF1* mRNA, 4 h following RE in older women (>80 y.o.) (Raue et al., 2007). However, in line with our findings, another study comparing similarly aged women to the study by Raue observed no changes in *MURF1* and atrogin-1 mRNA after 2.5 h of RE (Greig et al., 2011). Again, different population cohorts and exercise protocols may lead to different or temporally shifted gene expression patterns.

None of the proteins measured in this study displayed changes in response to a single-bout of RE in any of the age groups. The effects of single-bout RE on the protein levels and activity of FOXO1 and FOXO3 have only been partly described and phosphorylation of FOXO3 was unaltered at 1 h following RE (Glynn et al., 2010). However, MURF1 protein has been reported to slightly increase at 1 h following bilateral leg extension exercise (10 × 10 repetitions at 70% 1 RM) (Glynn et al., 2010) and at 3 h after unilateral leg press RE (4 × 10 repetitions at 80% followed by 4 × 15 at 65% 1 RM) (Borgenvik et al., 2012) in untrained younger subjects; a difference that might be explained by the lower intensity and total volume of work completed in our study. Consistent with this result, the expression levels of the MURF1 substrate targets PKM2 and MHC did not vary with exercise. While atrogin-1 mRNA was induced by exercise, this increase was not paralleled by a decrease in its substrate target proteins MYOD, myogenin and EIF3F. A lack of visible change in protein expression at 2 h post-exercise does not reflect a lack of functional outcome. With respect to the delay existing between gene transcription and protein translation, changes in protein levels might occur later than 2 h post-exercise, as reported with certain types of exercise protocols (Borgenvik et al., 2012). MPB measured after a single-bout of RE has been shown to be maximal at 3 h post-exercise (Phillips et al., 1997), suggesting that an increase in the proteolytic activity of MURF1 and atrogin-1 might occur concomitantly. In addition, E3-ubiquitin-ligases are reported to self-ubiquinate, so that protein levels may not always mirror mRNA levels (Sandri, 2013).

We report a decrease in FBXO40 gene and protein levels in the muscle of older subjects when compared to younger subjects. Similar to atrogin-1, *FBXO40* is a gene encoding a muscle specific F-box protein (Ye et al., 2007). F-box proteins associate with other proteins to form Skp1–Cullin1–F-box (SCF) complexes that regulate proteasome-mediated protein breakdown via their ubiquitin ligase activity. In skeletal muscle, FBXO40 ubiquitinates and degrades insulin receptor substrate 1 (IRS1); the latter being an upstream positive regulator of the PI3K/Akt pathway activity (Shi et al., 2011). Mice lacking FBXO40 demonstrate abnormal skeletal muscle hypertrophy. However, in humans, *FBXO40* gene expression decreases in the muscle of Limb-girdle muscular dystrophy (LGMD) patients (Ye et al., 2007). Attenuated FBXO40 expression in the skeletal muscle of older subjects might therefore reflect a compensatory mechanism to limit the amount of age-related muscle wasting. It would be of interest to determine if a resistance training intervention, potentially combined with amino acid supplementation, would be able to rescue, at least partially, the levels of FBXO40 in old muscle. Although we observed no age-related change in MURF1 protein levels, PKM2 protein expression tended to be higher in the older subjects when compared to younger subjects. PKM2 is a substrate target for MURF1 (Hirner et al., 2008) and a molecular switch toward a more glycolytic phenotype fiber type (Gao and Cooper, 2013). A possible hypothesis is that elevated PKM2 protein levels might account for the reduced oxidative metabolism associated with muscle wasting in older people (Rogers et al., 1990; Nair, 2005; Short et al., 2005), even if oxidative metabolism or fiber type composition have not been assessed in this study; however, PKM2 regulation is probably multi-factorial.

In conclusion, we demonstrate that the UPP-related genes involved in MPB display comparable regulation in healthy younger and older subjects following RE. However, the lack of significant difference in the physiological characteristics, body composition and level of fitness of our older subjects when compared to younger subjects must be considered when interpreting these findings. We did not observe a change in the baseline levels of atrogin-1 and MURF1 mRNA and protein of younger vs. older individuals, but identified an age-related decline in FBXO40, another recently described muscle specific F-box protein. Our observations support the hypothesis that the UPP plays a limited role in the disruption of the protein synthesis/degradation balance that is characteristic of age-related muscle atrophy. However, future investigations are required to measure skeletal MPB following RE in the ageing population. Additionally, measurements of proteasomal content and/or proteasomal activity as well as investigation of the role of other protein degradation mechanisms, such as the autophagy-lysosome system, will aid in gaining a deeper understanding of the contribution of the ubiquitin-proteasome system in anabolic resistance.

### Conflict of interest statement

The authors declare that the research was conducted in the absence of any commercial or financial relationships that could be construed as a potential conflict of interest.

## Footnotes

^1^http://www.wma.net/en/30publications/10policies/b3/
